# The ‘Swallow Tail’ Appearance of the Healthy Nigrosome – A New Accurate Test of Parkinson's Disease: A Case-Control and Retrospective Cross-Sectional MRI Study at 3T

**DOI:** 10.1371/journal.pone.0093814

**Published:** 2014-04-07

**Authors:** Stefan T. Schwarz, Mohammed Afzal, Paul S. Morgan, Nin Bajaj, Penny A. Gowland, Dorothee P. Auer

**Affiliations:** 1 Radiological Sciences, Division of Clinical Neurosciences, School of Medicine, University of Nottingham, Nottingham, United Kingdom; 2 Department of Medicine, Nottingham University Hospitals NHS Trust, Nottingham, United Kingdom; 3 Medical Physics, Nottingham University Hospitals NHS Trust, Nottingham, United Kingdom; 4 Department of Neurology, Nottingham University Hospitals NHS Trust, Nottingham, United Kingdom; 5 Sir Peter Mansfield Magnetic Resonance Centre, School of Physics and Astronomy, Nottingham, United Kingdom; University of Melbourne, Australia

## Abstract

There is no well-established *in vivo* marker of nigral degeneration in Parkinson's disease (PD). An ideal imaging marker would directly mirror the loss of substantia nigra dopaminergic neurones, which is most prominent in sub-regions called nigrosomes. High-resolution, iron-sensitive, magnetic resonance imaging (MRI) at 7T allows direct nigrosome-1 visualisation in healthy people but not in PD. Here, we investigated the feasibility of nigrosome-1 detection using 3T - susceptibility-weighted (SWI) MRI and the diagnostic accuracy that can be achieved for diagnosing PD in a clinical population. 114 high-resolution 3T – SWI-scans were reviewed consisting of a prospective case-control study in 19 subjects (10 PD, 9 controls) and a retrospective cross-sectional study in 95 consecutive patients undergoing routine clinical SWI-scans (>50 years, 9 PD, 81 non-PD, 5 non-diagnostic studies excluded). Two raters independently classified subjects into PD and non-PD according to absence or presence of nigrosome-1, followed by consensus reading. Diagnostic accuracy was assessed against clinical diagnosis as gold standard. Absolute inter- and intra-rater agreement was ≥94% (kappa≥0.82, p<0.001). In the prospective study 8/9 control and 8/10 PD; and in the retrospective study 77/81 non-PD and all 9 PD subjects were correctly classified. Diagnostic accuracy of the retrospective cohort was: sensitivity 100%, specificity 95%, NPV 1, PPV 0.69 and accuracy 96% which dropped to 91% when including non-diagnostic scans (‘intent to diagnose’). The healthy nigrosome-1 can be readily depicted on high-resolution 3T - SWI giving rise to a ‘swallow tail’ appearance of the dorsolateral substantia nigra, and this feature is lost in PD. Visual radiological assessment yielded a high diagnostic accuracy for PD vs. an unselected clinical control population. Assessing the substantia nigra on SWI for the typical ‘swallow tail’ appearance has potential to become a new and easy applicable 3T MRI diagnostic tool for nigral degeneration in PD.

## Introduction

Parkinson's disease (PD) is a progressive neurodegenerative disorder resulting in characteristic motor and non-motor symptoms. The motor features form the basis of the clinical diagnosis and are embedded in the UK-Brain bank criteria for diagnosing PD [1]. Establishing the clinical diagnosis can be challenging in early or tremor dominant cases. The reported diagnostic error rates are 4-15% in clinical trials and up to 25% in community studies [2]–[4]. Ioflupane (123I) single photon emission computer tomography (SPECT) has proved useful in establishing presynaptic dopaminergic loss in difficult diagnostic cases [5] using dopamine transporter imaging of the striatum as a surrogate marker for nigral dopaminergic cell loss. This technique has been recently approved by the American Food and Drug Administration for diagnostic purposes in PD (DaTscan, approved January 2011). However, these types of scans are expensive, involve low dose radiation and are only available in specialised centres. Correlation with disease progression is limited. Furthermore, DaTscan is not currently licensed for differentiation of some of the parkinsonian conditions that can be mistaken for PD, including progressive supranuclear gaze palsy parkinsonian variant (PSP-P) and multiple system atrophy type P (MSA-P) [6].

Research into alternative biomarkers of PD in recent years has demonstrated a range of genetic, biochemical and also imaging techniques with varying ranges of accuracy, repeatability and reliability [7]. However, to date, none of the investigated methods demonstrate the required accuracy and ease of use to allow translation to standard clinical practice and DaTscan remains the only licensed PD diagnostic tool.

The neurons of the pars compacta of the substantia nigra (SN_PC_) are affected early in PD, with loss of 60-80% of neurons described before manifestation of motor symptoms [8]. Nigrosomes represent small clusters of dopaminergic cells within the healthy substantia nigra exhibiting calbindin D_28K_ negativity on immunohistochemical staining [9]. The largest nigrosome of the five described nigrosomes is labelled nigrosome-1 and positioned in the caudal and medio-lateral SN. It contains the biggest proportion of neurons which are most commonly affected by PD [10]. Despite the small physical size of only a few mm, both the healthy nigrosome-1 and PD induced pathological change within nigrosome-1 can be demonstrated with high resolution T2*/SWI (HR-SWI) weighted magnetic resonance imaging (MRI) at ultra-high magnetic field strengths of 7T [11].

Translating this technique to 3T - MRI platforms in order to support the diagnosis of PD would be highly desirable, as MRI at 3T is widely available and much less expensive than licensed nuclear medical techniques. It additionally offers the opportunity for combining diagnostic confirmation of nigral degeneration in PD with promising MRI techniques for differentiating other parkinsonian conditions such as PSP-P, MSA-P and vascular parkinsonism [12], [13]. In this combined prospective and retrospective study including 114 SWI scans at 3T, we investigated the feasibility of HR-SWI MRI at 3T to depict PD related signal loss within nigrosome-1 and the diagnostic accuracy that can be achieved in a clinical population (50+ years of age).

## Methods

### Prospective case-control study

This study was part of a multimodal MRI characterization of the substantia nigra in patients with PD that was approved by the local Research Committee (North Nottinghamshire Research Ethics Committee, UK, reference 05/Q2402/87) and the Trust Research and Development Department (Nottingham University Hospitals NHS Trust) [14], [15]. Written informed consent was obtained from all participants in person before enrolment in the study. Assessment of capacity and obtaining of consent was performed by a neurology consultant of the University of Nottingham Hospitals NHS trust. Ten patients fulfilling the UK Brain Bank criteria for PD were recruited from movement disorders clinics at two local National Health Service trusts. Nine age-matched controls were recruited from spouses and friends. All subjects underwent the Addenbrooke's Cognitive Examination (ACE) test battery [16] and had to have a score >80 for inclusion. All participants had Mini-Mental State examination scores ≥27 [17]). Disease severity was recorded using the UPDRS [18] and Hoehn and Yahr clinical rating scales for PD [19]. The patients were sub-categorized into tremor dominant cases (TDPD) and postural instability and gait disturbance patients (PIGD) according to previously published assessment methods [20]. Patients were assessed and scanned whilst medicated.

MR imaging was performed at 3T (Achieva scanner, Philips Medical Systems, Best, Netherlands) with a standard eight-channel head coil. The image acquisition was performed using a HR-SWI weighted sequence with 3D acquisition using a gradient echo planar imaging sequence (FEEPI, TR/TE 60/30, echo train length 5, Flip angle 19°, number of slices: 70, voxel size 0.55×0.55×0.7 mm, scan duration: 4 minutes 26 seconds, only magnitude image used). The orientation of the axial slices was individually aligned parallel to the splenium and genu line of the corpus callosum. The magnitude images were reviewed in multiple planes using 3D slicer or a locally developed image viewing and analysis software (NeuRoi, http://www.nottingham.ac.uk/research/groups/clinicalneurology/neuroi.aspx) on standard PC-LCD flat screen displays.

### Retrospective cross-sectional study

The retrospective study was performed by clinical personnel as part of an institutionally approved service evaluation/audit (Department of Radiology, Nottingham University Hospitals NHS Trust) to assess the potential of improved patient care after introduction of a HR-SWI sequence into routine MRI protocols. As per local guideline approval was obtained from the Directorate Clinical Director and Head of Neuroradiology Service who in accordance of the Caldicott guardian waived informed consent for the retrospective data collection and review. We also obtained guidance by a chair holder of the Research Ethics Committee (Nottingham Research Ethics Service, Health Research Authority, UK) who confirmed that there is no requirement for a formal Research Ethics Committee review.

The imaging data were reviewed on local radiology reporting workstations (AGFA IMPAX 6.5.2.657, 3D reconstructions: AGFA IMPAX Volume Viewing 2.2, Clinapps 4.2.31, AGFA Healthcare, Mortsel, Belgium). Patient clinical data were collected by retrospectively reviewing patient clinical information (Nottingham information system – NOTIS). 105 consecutive patients undergoing HR-SWI as part of their scanning protocol (data recording period October 2012 – May 2013) were assessed. Of these, nine patients under the care of trust specialty neurology consultants were previously diagnosed with PD in accordance to UK Brain Bank Criteria. In another six cases the patient's clinical symptoms raised the possibility of PD, but no formal UK Brain Bank diagnosis was established, hence patients were labelled as unclear movement disorder diagnosis and excluded from analysis. We also excluded patients with structural brainstem pathology resulting in distortion of the substantia nigra (n = 4). A total of five scans showed severe artefact due to patient movement preventing the assessment of the presence of nigrosome-1. This resulted in a retrospective cohort of 90 cases including 9 clinical PD and 81 patients with clinical diagnoses other than PD without any documented movement disorder symptoms suggestive of parkinsonism (Table S1 in File S1). We additionally describe the sensitivity/specificity analysis when including the 5 non-interpretable scans (n = 95 cases for ‘intent to diagnose’ analysis).

In the retrospective study, MR imaging was performed on the same scanner as above, also with a standard eight-channel head coil, using a standard clinical Phillips ‘Principles of Echo Shifting with a Train of Observations' (PRESTO) sequence. PRESTO is a HR-T2*/SWI 3D multi-shot fast field echo – echo planar imaging sequence FFE-EPI. TR/TE 16.55/23.29, echo train length 1, Flip angle 10°, number of slices: 200, voxel size 0.43×0.43×0.75 mm^3^, SENSE factor 2, Scan duration: 2 minutes 36 seconds). The orientation of acquired axial slices was in alignment with the splenium and genu of corpus callosum in the sagittal plane.

### Nigrosome-1 depiction and ‘swallow tail’ appearance

MRI scans were visually assessed for absence or presence of nigrosome-1 hyperintensity independently by two blinded neuroradiologists with a special interest in neurodegenerative diseases and more than 20 and 5 years of clinical and research neurological MRI experience respectively. Scan quality, especially in clinical scans, can vary and therefore retrospective study MRI scans were assessed and scored for scan quality (for details on quality assessment, see supplementary material: MRI scan quality assessment).

**Figure 1:**
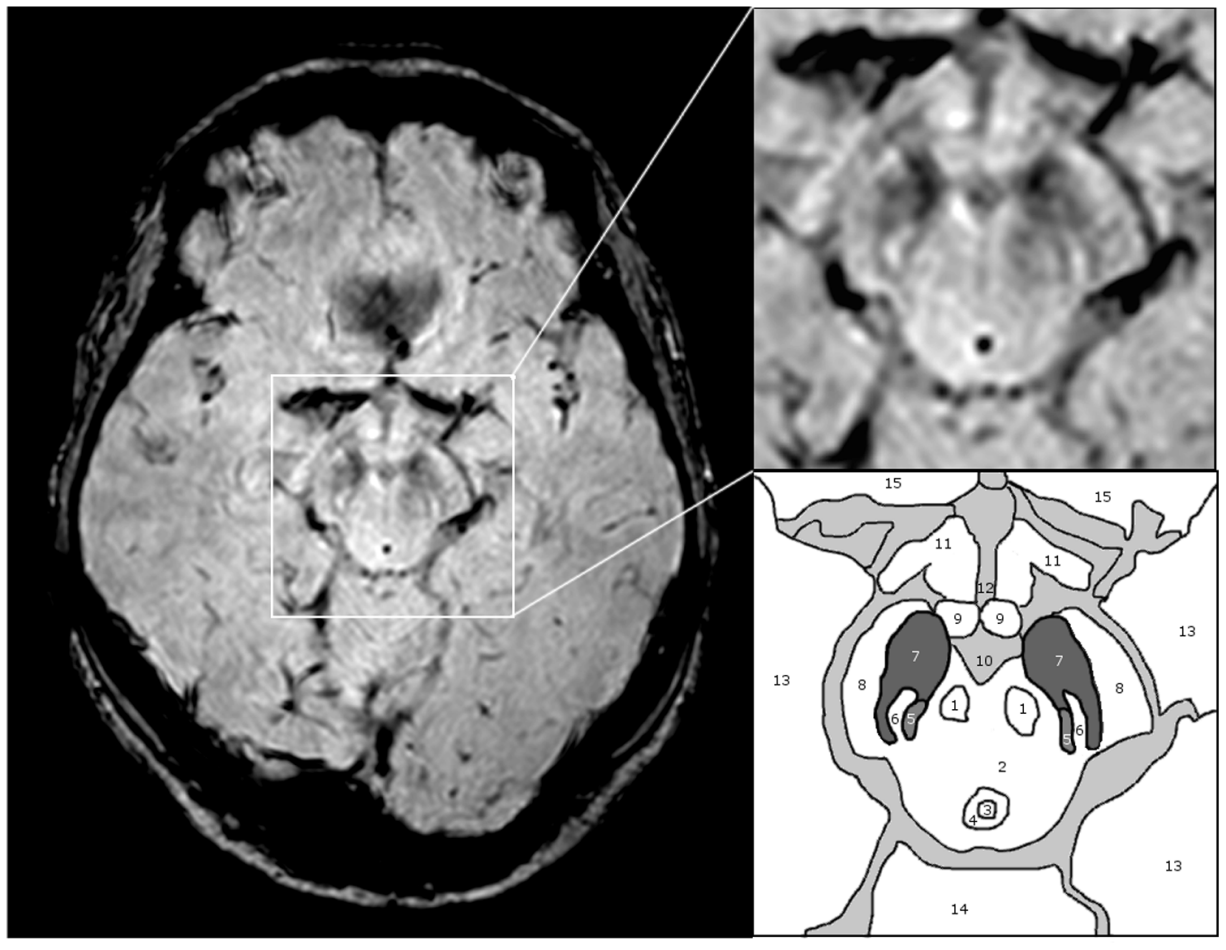
Substantia nigra anatomy on 3T - SWI – MRI. Demonstrated is a 3T - SWI axial slice just at the level of nigrosome-1 with magnification of the midbrain structures and a sketch outlining relevant anatomical structures: 1 red nucleus, 2 midbrain tegmentum, 3 aqueduct, 4 periaqueductal grey, 5 medial leminiscus, 6 nigrosome-1, 7 substantia nigra, 8 cerebral peduncle, 9 mammillary body, 10 inter-peduncular fossa, 11 optic radiation, 12 3^rd^ ventricle, 13 temporal lobe, 14 cerebellum, 15 frontal lobe.

**Figure 2:**
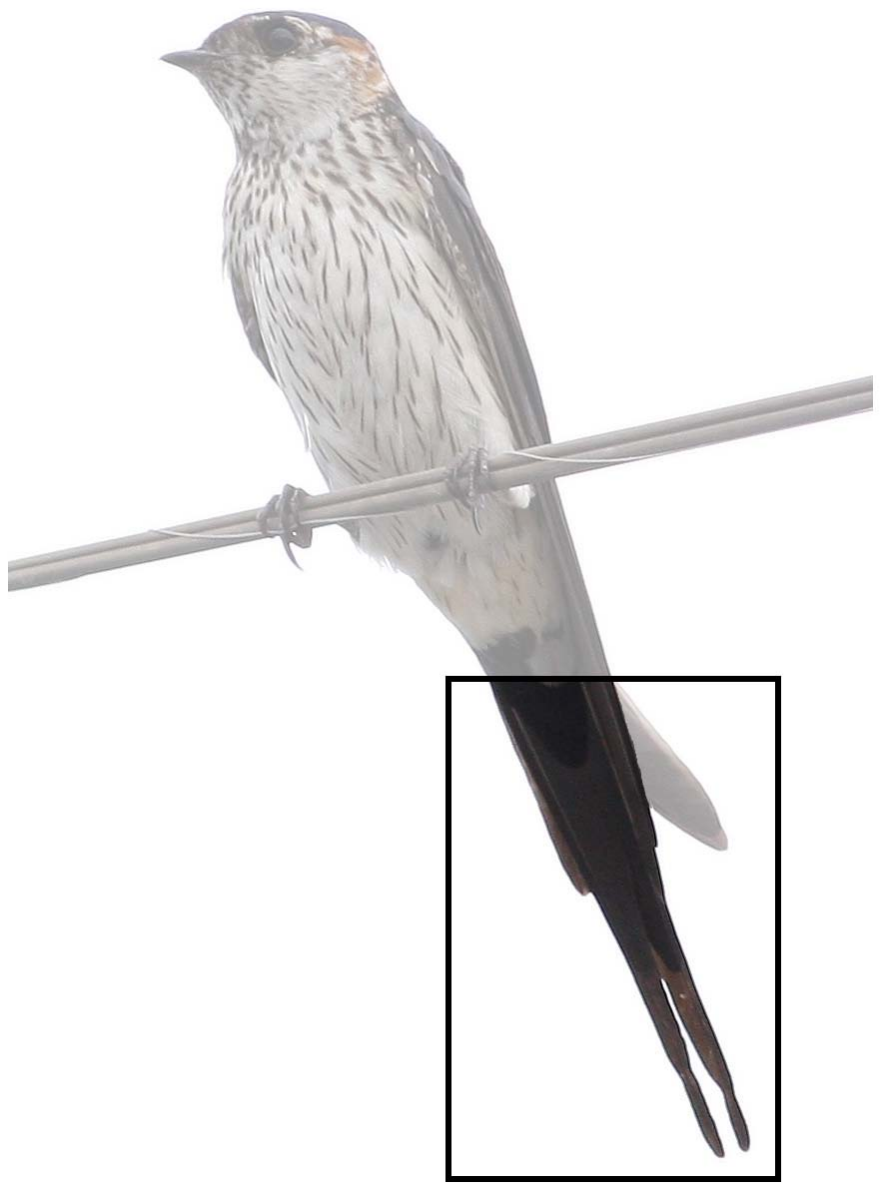
The red rumped swallow (Cecropis daurica). The tail of the swallow resembles the appearance of the healthy nigrosome-1 on HR-SWI MRI. Picture modified from Wikipedia: http://en.wikipedia.org/wiki/Swallow. Image rights transferred to public domain.

Nigrosome-1 is located in the posterior third of the substantia nigra, returns a high signal on SWI in a ‘linear’, ‘comma’ or ‘wedge’ shape and is surrounded by low SWI signal intensity anterior and laterally (pars compacta SN) and medially by low signal from the medial leminiscus (Fig. 1). Nigrosome-1 and its surrounding structures have resemblance to the tail of a swallow on axial imaging HR-SWI (Fig. 1 and Fig. 2).

**Figure 3:**
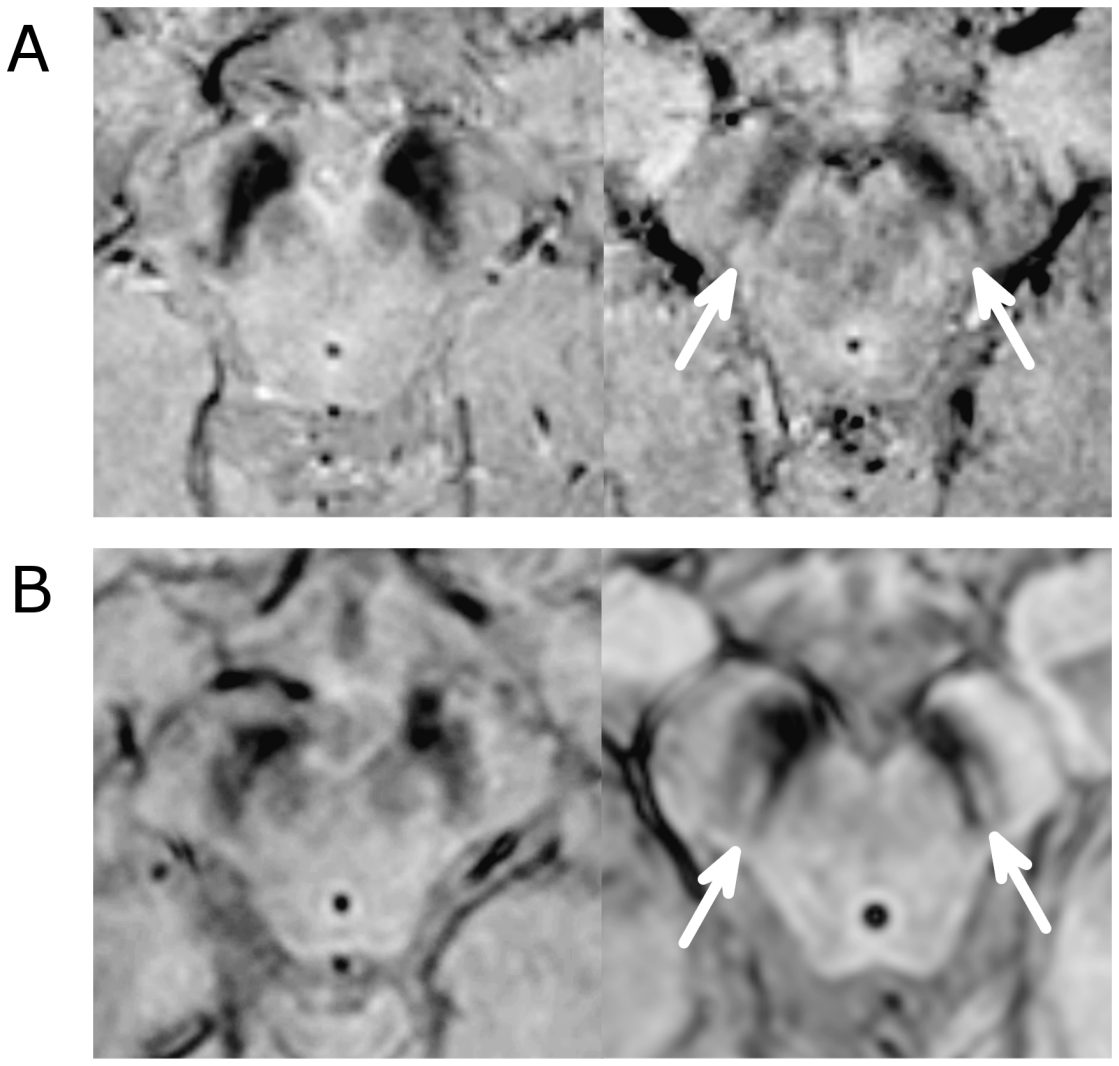
SWI MRI in PD and Non-PD patients. A. High resolution SWI MRI (3D gradient echo EPI, magnitude image) of a PD patient (left, 60 years, female, UPDRS: 53, HY score 3, nigrosome-1 absent bilaterally) and a control (right, 61 years, female, nigrosome-1 present bilaterally). B. Clinical high resolution 3D-T2*/SWI MRI (Philips ‘PRESTO’ sequence), of a PD patient (left, 58 years old, male, nigrosome-1 absent bilaterally) and a non-PD patient (right, 70 years old, female, diagnosed with an aneurysmal subarachnoid haemorrhage, nigrosome-1 present bilaterally).

Presence of the swallow tail/nigrosome-1 was rated for each hemi-mesencephalon at the level of the caudal posterior SN on axial and reformatted scans by both raters individually. Nigrosome-1 scores allowed were present, absent or ‘possibly present’. Given the asymmetrical onset of PD, unilateral absence of nigrosome-1 was classified as indicative of PD irrespective of the presence or absence of nigrosome-1 on the other side. Scans were therefore classified into three groups taking into account nigrosome-1 assessment. Group I: ‘normal’ (nigrosome-1 present bilaterally - or - unilateral nigrosome-1 present and contralateral ‘possibly present’), Group II: ‘non diagnostic’ (bilateral possibly present), Group III: ‘abnormal’ (nigrosome-1 absent unilateral or bilateral, see also Fig. 3). Reproducibility of nigrosome scoring for Inter- and Intra-rater (>4 weeks interval between image analysis) variability was tested by calculation of absolute and kappa statistics. Consensus agreement for cases which were scored differently by the two investigators was sought in a final assessment.

### Statistical Analysis, Image generation and reporting guidelines

Statistical tests were performed using IBM SPSS for windows (version 19.0) and Microsoft Excel 2010. Demographics were compared between patients and controls using analysis of variance. Non-parametric tests were used for group comparisons in case of non-normal distribution of the data. Values are given as mean ± SD unless stated otherwise; significance was defined at p<0.05. If applicable, sample distribution was assessed by Fisher's exact test. Images for illustration were generated using 3D slicer (http://www.slicer.org)[21] and GNU Image Manipulation Program (GIMP V2.8, http://www.gimp.org). QUADAS-2 and STARD recommendations were used as a guide for analysis and reporting the study outcomes [22], [23].

## Results

### Inter- and Intra-rater agreement of nigrosome-1 assessment

102/109 cases were classified into the same groups by both raters (109 consisting of 19 prospective study and 90 retrospective study subjects). Absolute inter-rater agreement was 94% (kappa = 0.82, p<0.001) and intra-rater agreement 94% (kappa = 0.82, p<0.001).

### Prospective case-control study

PD patients and controls did not differ in age or sex (PD: 68.2±11 years, 3 males vs. controls: 66.4±7.6 years, 6 males). Disease severity of PD patients ranged from mild to moderate (UPDRS = 8 to 60, mean ± SD: 32.5±15.4, HY = 1 to 3, mean ± SD: 1.85±0.9, PIGD = 7, TDPD = 3) with a disease duration of 4±3.4 years (range: 1 to 10 years). No cases were excluded from the analysis due to poor quality or movement artefact. 16 subjects (8 PD and 8 controls) were correctly classified by both raters independently (Fig. 3a). Three subjects (2 PD and 1 control) were misclassified (1 PD patient after consensus agreement) yielding a consensus diagnostic accuracy of 84% (sensitivity: 80%, specificity 89%, see also Table S2 in File S1).

### Retrospective cross-sectional study

**Figure 4:**
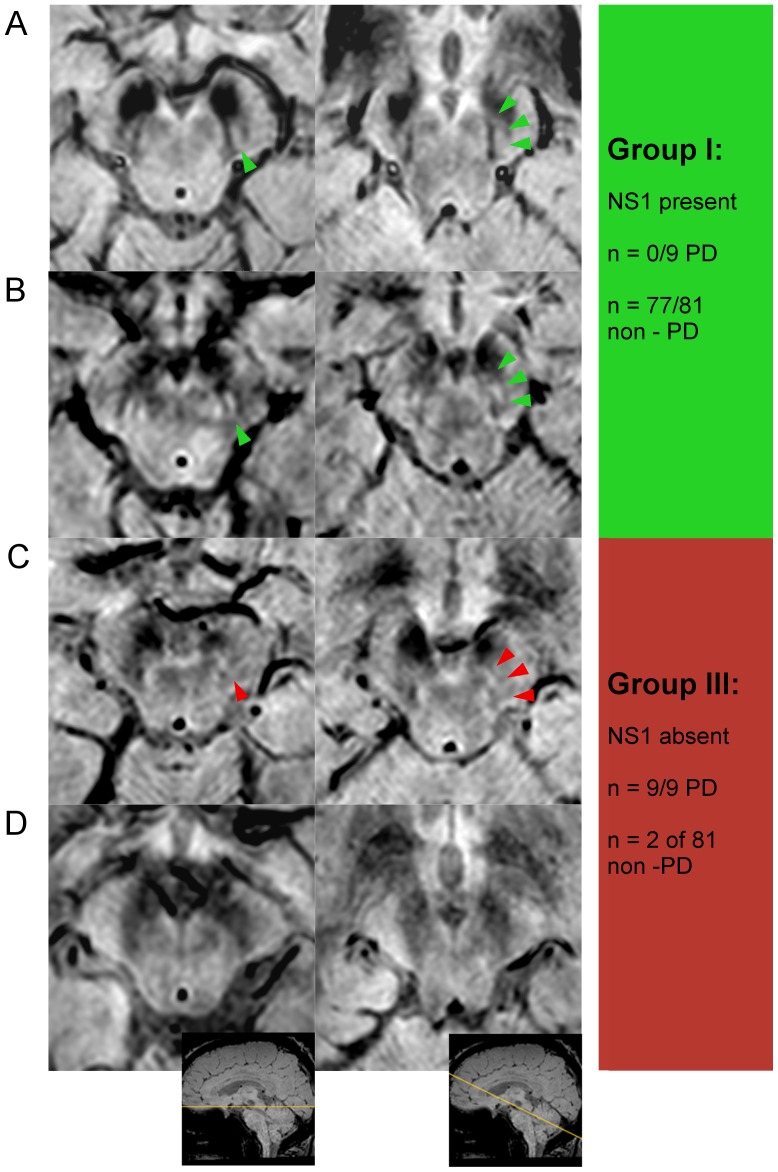
Clinical HR-SWI MRI at 3T (‘PRESTO’ sequence) to demonstrate nigrosome-1. Nigrosome-1 assessment of the SN in 4 different patients (a-d). The first column illustrates the standard axial plane at the level just inferior to red nucleus and the second column the multi-planar reformat along the axis of the low density signal of the SN (oblique axial). Green arrow tip illustrates nigrosome-1 presence; red arrow tip illustrates unclear/possible presence of nigrosome-1. a) Non-PD patient (60 years old, female, diagnosed with cognitive impairment and a small cerebral cavernoma) – nigrosome-1 is present bilaterally. b) Non-PD patient (54 year old, female, MRI scan for recurrent loss of consciousness of unknown cause) – nigrosome-1 is present bilaterally but smaller than in (a). c) PD patient (68 years old, male) - Right nigrosome-1 absent, left nigrosome-1 unclear. d) PD patient (65 years old, female) – nigrosome-1 absent bilaterally. An example of Group II (bilateral unclear/possible nigrosome-1 presence, n = 2 of 81 non-PD patients) is not demonstrated.

90 consecutive clinical cases (>50 years of age, mean 66.3±10.6 years, range 50–89.5 years, 49 males) underwent nigrosome-1 review as described above. Both raters considered the quality (QR - see also supplement methods in File S1) as similar between group I and III (QR group I: 1.79±0.62, QR group III: 1.7±0.62, n.s.). Two of 90 patients were classified into group II (Possible presence of nigrosome-1/uncertain scans). Out of 90 subjects, the same 81 (9 PD and 72 non-PD) were correctly classified by both raters independently. 86 of 90 patients were correctly classified (all 9 PD and 77 non-PD) based on consensus rating with similar results for independent rating (Fig. 3b and 4). Two of the 81 non-PD patients were wrongly classified as nigrosome-1 absent (group III). Table 1 demonstrates the sensitivity/specificity analysis for both raters individually and after consensus review of discrepantly scored cases (consensus diagnostic accuracy: 96%, sensitivity: 100%, specificity 95%).

**Table 1:** Analysis of diagnostic accuracy of nigrosome-1 presence to diagnose PD in a clinical population (non-diagnostic cases excluded).

N = 90	Rater A	Rater B	Consensus
**Sensitivity**	100%	100%	100%
**Specificity**	91%	93%	95%
**Pos. Pred. Val.**	56%	60%	69%
**Neg. Pred. Val.**	100%	100%	100%
**Accuracy**	92%	93%	96%

Analysis of diagnostic accuracy of nigrosome-1 presence to diagnose PD for each rater individually and after consensus assessment of discrepantly rated cases. The cases were classified according to presence or absence of nigrosome-1 and include nine PD cases and 81 non-PD cases (n = 90).

#### Intent to diagnose analysis of retrospective cross-sectional study cases

To avoid an inflated diagnostic accuracy resulting from pre-selection based on diagnostic quality, we also assessed sensitivity/specificity in the cohort of n = 95 (5 non-diagnostic investigations added to the above 90 cases) which were intended to be assessed for diagnostic purposes. This reduced the consensus diagnostic accuracy to 91% (sensitivity: 100%, specificity 90%, Table 2).

**Table 2:** Analysis of diagnostic accuracy of nigrosome-1 presence to diagnose PD in a clinical population with all non-diagnostic cases included.

(N = 95, all intended to diagnose)	Rater A	Rater B	Consensus
**Sensitivity**	100%	100%	100%
**Specificity**	86%	87%	90%
**Pos. Pred. Val.**	43%	45%	50%
**Neg. Pred. Val.**	100%	100%	100%
**Accuracy**	87%	88%	91%

Analysis of diagnostic accuracy of nigrosome-1 presence to diagnose PD for each rater individually and after consensus assessment of discrepantly rated cases. The cases were classified according to presence or absence of nigrosome-1 and include the 90 cases from Table 1 and five non-diagnostic scans (n = 95, ‘intended to diagnose cases’).

## Discussion

We demonstrate that standard high resolution susceptibility weighted MRI at 3T allows the detection of the healthy nigrosome-1, and its absence in PD, yielding a high diagnostic accuracy in a clinical population.

The sub-classification of the substantia nigra pars compacta (SNpc) into nigrosomes and the nigral matrix according to specific immunohistochemical staining properties was first described by Damier in 1999 [9]. Calbindin D_28K_ is the antibody used to demonstrate calbindin, a protein present in striato-nigral afferent fibres. According to calbindin presence, the SNpc was subdivided into the nigral matrix (calbindin rich) from the nigrosomes (calbindin poor). Sixty per cent of all dopamine containing neurons were found in the nigral matrix and 40% in five different nigrosomes (nigrosome-1 to nigrosome-5). Even though the definite functional relevance of this subdivision remains unclear, Damier found that the largest of the nigrosomes, positioned in the ventro-lateral substantia nigra (nigrosome-1), was most affected in PD exhibiting the maximum depletion of dopaminergic cells of 98% [10].

Using high-resolution SWI MRI at 3T, we were able to reliably visualize the nigrosome *in vivo*. The appearance of the dorsolateral SN on standard axial high resolution susceptibility weighted MRI in healthy controls and in non-PD patients has remarkable similarity to the tail of a swallow. This is due to the presence of a high signal intensity wedge previously shown to represent the nigrosome-1 [11]. Visibility of nigrosome-1 is enhanced by subtotal outlining of low intensity structures on T2* images allowing for 3D reconstructions of the wedge-like nigrosome on 7T - T2* [24]. Direct depiction of SN subcomponents is a new concept which was very recently introduced using sub-millimetre ultrahigh-field MRI that allowed detection of the previously unknown intrinsic contrast mechanisms. Earlier MRI studies in PD are limited as the boundaries of the substantia nigra pars compacta and pars reticulata are not well defined on MRI. This is a recognised source of heterogeneity between studies reporting quantitative MRI properties of SN pathology [25].

Intriguingly, the swallow tail sign was absent in patients with PD, both in the case-control study and the retrospective cross-sectional clinical population study. In the cross-sectional study we found the diagnostic accuracy ranging between 96% in the diagnostic scans and 91% in all scans with ‘intent to diagnose’. These findings concord well with the small case-control study at 7T - MRI [11], proving successful translation of the preliminary 7T finding to a clinically widely used 3T - MRI platform.

Inability to visualise nigrosome-1 on HR-SWI MRI does not mean nigrosome-1 is completely lost, but that the signal increase compared to its surround is lost. While we cannot exclude a degree of nigrosome volume loss, the main effect can be explained by signal loss of the hyperintense nigrosome-1 (structure 6 in Fig. 1) in comparison to the adjacent low signal substantia nigra (structure 7 in Fig. 1) and low signal medial leminiscus (structure 5 in Fig. 1) resulting in homogenous dark appearances. There are two main possible mechanisms for this, increased iron content or decreased neuromelanin content with decreased iron storage capacity leading to more free iron with paramagnetic properties [26]. Previous MRI studies at 3T or 1.5T assessing iron related PD induced nigral changes focussed on differences in the whole SN [27], attempted subdivision of the SN into pars reticulata and pars compacta [28] or used a voxel based morphometric analysis approach [29]. These studies confirm significant iron related SN signal alterations on group level, some of which even show correlation to disease severity measures like UPDRS. However, the highest resolution study [29] uses voxel sizes of 1×1×2 mm^3^ which is inappropriate for reliable assessment of small SN substructures like nigrosome-1. Voxel sizes in the sub-millimetre region, as used in our 3T MRI study, proved sufficient to reliably delineate the healthy nigrosome-1.

In our retrospectively studied clinical population, the nigral swallow tail sign was associated with a negative predictive value of 100% indicating that nigrosome-1 presence was associated with a very low likelihood of PD. In 2% of the non-PD patients over 50 years of age, the nigrosome was not detectable. This rate is similar to the rate of prevalence of PD in the elderly [30] indicating a possibility of undiagnosed PD in these cases. 5/95 clinical cases were of poor non-diagnostic quality (caused by patient movement) prohibiting the assessment for the presence of a swallow tail appearance. As the scan lasts less than 3 minutes, standard measures to reduce patient movement during studies, like scan repetition after patient prompting or additional fixation of the patient's head within the head coil, are likely to increase the rate of scans of diagnostic quality. Some cases may require light sedation. The observed rate of non-diagnostic scans without these additional measures of less than 5.2% compares favourably with a rate of 5–20% failed transcranial ultrasound imaging investigations for PD [31].

In current clinical practice suspected PD can be differentiated from non-parkinsonian movement disorders like benign tremor with licensed nuclear medical techniques using e.g. ^123^ioflupane (^123^I, ^123^I-FP-CIT single photon emission computed tomography (SPECT) or DaTscan) to demonstrate presynaptic dopamine transporter loss with up to 95% accuracy [6]. Even though our control group was chosen not to include patients with uncertain movement disorders, the comparable high sensitivity/specificity obtained to diagnose PD vs. non PD based on nigrosomal signal abnormality highlight the diagnostic potential of 3T SWI scans. Nuclear medical scans have intrinsic associated high costs, cause radiation exposure and access to these types of scans is limited in many countries in contrast to widespread availability of MRI. A MRI based diagnostic test for PD would have the further advantage that it can be combined with standard MRI to exclude focal pathology, assess vascular causes and comorbidity as well as regional degenerative markers of other atypical parkinsonian syndromes [32]. Advanced MRI techniques including volumetry and diffusion tensor imaging (DTI) metrics of midbrain, cerebellum and pons show promise to aid the differential diagnosis of atypical parkinsonian syndromes for 1.5T MRI systems with as yet limited evidence for 3T MRI- DTI systems [33].

We found that the swallow tail appearances of the healthy nigrosome can be readily and reliably recognised by trained radiologists making it an attractive diagnostic imaging sign. This is clearly advantageous for clinical workflow over previously proposed MRI markers requiring dedicated postprocessing which may have contributed to inconsistent results. Nigral diffusion metrics were reported as promising diagnostic tool for PD but lack consistency as shown in a recent meta-analysis [25]. Other MRI based techniques especially iron and neuromelanin sensitive MRI hold promise to track PD severity [14],[34] but as yet require dedicated postprocessing and/or subjective regional measurements which introduce user dependency. This limits the reliability and utility of these techniques in standard clinical practice or as part of research trials. In this study, standard high resolution T2*/SWI sequences were used for image acquisition in a small prospective study population but also in a larger standard clinical population. There was no requirement for complicated post processing prior to visual assessment for presence or absence of nigrosome-1 which confirms ease of usability and attractiveness of this technique for everyday clinical use.

Even though the patients of the prospective arm of this study were clinically well characterised, only limited clinical information was available for the retrospective study of patients. Even if the likelihood of undiagnosed PD patients within the non-PD group of the retrospective study is low (2% in >60 year olds and majority of patients receive follow up and treatment for a non-PD related condition in our institution), we cannot exclude the possibility that a small number of ‘non-PD classified' patients have preclinical PD. In the context of biases of accuracy studies this could be considered as a ‘disease spectrum bias’ and could potentially falsely raise the sensitivity of the proposed test [35].

The avoidance of a case-control design and the retrospective study approach to assess diagnostic accuracy is in accordance to guidance from QUADAS-2 (Revised Tool for the Quality Assessment of Diagnostic Accuracy studies [23]). However, in the retrospective study arm we had to exclude cases with clinical movement disorder type symptoms but no definitely established diagnosis of PD (n = 6 of 105 patients) and our PD patient cohort only included patients with clinically established PD. Therefore we are unable to comment on the timing of nigrosome changes in relation to the onset of parkinsonian symptoms and disease severity measures. Longitudinal studies of subjects with MR absent nigrosomes will allow to investigate the predictive value of this technique in populations at high risk of developing PD. Independent prospective validation studies are needed to assess the trajectory of MRI nigrosome-1 changes in relation to the pre-motor and motor stages of PD and to correlate nigrosome-1 changes with loss of presynaptic dopamine transporters as demonstrated by nuclear medical scan techniques such as DaTscan.

## Conclusion

The healthy nigrosome-1 can be readily depicted on high-resolution 3T - SWI giving rise to a ‘swallow tail’ appearance of the dorsolateral substantia nigra, and this feature is lost in PD. Visual radiological assessment yielded a high diagnostic accuracy for PD vs. an unselected clinical control population. Assessing the substantia nigra on SWI for the typical ‘swallow tail’ appearance has potential to become a new and easy applicable 3T MRI diagnostic tool for nigral degeneration in PD.

## Supporting Information

File S1 **Supporting methods, Tables S1 and S2.** Methods: MRI scan quality assessment. Table S1: ‘Leading’ diagnosis/symptoms of patients included in the retrospective review of high resolution T2* weighted imaging of the SN at 3T. Table S2: Analysis of diagnostic accuracy of nigrosome-1 presence to diagnose PD in the prospective study.
(DOCX)
