# Supplementary material for: The ‘Swallow Tail’ Appearance of the Healthy Nigrosome – A New Accurate Test of Parkinson's Disease: A Case-Control and Retrospective Cross-Sectional MRI Study at 3T
Source: PLoS One. 2014 Apr 7;9(4):e93814. doi: 10.1371/journal.pone.0093814 (PMC3977922; doi:10.1371/journal.pone.0093814)
Supplement: File S1 — Supporting methods, Tables S1 and S2. Methods: MRI scan quality assessment. Table S1: ‘Leading’ diagnosis/symptoms of patients included in the retrospective review of high resolution T2* weighted imaging of the SN at 3T. Table S2: Analysis of diagnostic accuracy of nigrosome-1 presence to diagnose PD in the prospective study. (DOCX) [file pone.0093814.s001.docx]

# Supporting information/Appendix

## Methods: MRI scan quality assessment

SWI MRI is prone to artefacts due to vascular or CSF flow, patient movement or air/bone/brain interfaces from para-nasal sinuses and mastoid air cells. The clinical scans were performed on patients with a wide range of pathologies and scan duration with SWI usually at the end of the scanning session. Quality of scans was assessed as: 1 = very good (little or no artefact); 2 = good ( some artefact); 3 = poor (considerable artefact); 4 = very poor (significant artefact, just interpretable), 5 = non diagnostic scan.

## Table S1:

‘Leading’ diagnosis/symptoms of patients included in the retrospective review of high resolution T2* weighted imaging of the SN at 3T.

| Number of Patients | Diagnosis |
| --- | --- |
| 22 | Dementia (of Alzheimer’s type or FTD or dementia with non-specific generalized atrophy) |
| 16 | Cerebrovascular disease (CVA/TIA) |
| 7 | Developmental venous anomaly/cavernoma |
| 6 | Subdural haematoma, intracranial haemorrhage |
| 5 | Subarachnoid haemorrhage with or without intracranial aneurysm |
| 3 | Vestibular symptoms |
| 3 | Brain tumour/Brain metastasis |
| 3 | Encephalitis/brain abscess |
| 2 | Meningioma |
| 2 | Multiple Sclerosis |
| 2 | Cervical spine spondylosis with neck radiculopathy |
| 1 | Venous sinus thrombosis |
| 1 | Idiopathic intracranial hypertension |
| 1 | Vasculitis |
| 1 | Epiphora |
| 6 | Non-specific symptoms with no MRI changes and no specific diagnosis (Traumatic head injury, Transient global amnesia, headache/Migraine) |
|  |  |
| 9 | Parkinson’s disease |
|  |  |
| 5 | Severe artefacts due to patient movement casing inability to assess for nigrosome-1 |
|  |  |
| 10 | Excluded from analysis |
|  | N=4 Extensive abnormality causing severe anatomical distortion of the midbrain |
|  | N=6 Patients with non-definite diagnosis in the background of an unclear movement disorder |
|  |  |

Table S2:

Analysis of diagnostic accuracy of nigrosome-1 presence to diagnose PD in the prospective study

| N=19 | Rater A | Rater B | Consensus |
| --- | --- | --- | --- |
| Sensitivity | 90% | 80% | 80% |
| Specificity | 89% | 89% | 89% |
| (Pos. Pred. Val.) | (90%) | (89%) | (89%) |
| (Neg. Pred. Val.) | (89%) | (80%) | (80%) |
| Accuracy | 89% | 84% | 84% |

Analysis of diagnostic accuracy of nigrosome-1 presence to diagnose PD for each rater individually and after consensus assessment of discrepantly rated cases. Please note that the negative predictive value and positive predictive value of case-control accuracy studies are of limited value[30] as indicated by brackets*.*
